# Emergency Single-Incision Laparoscopic Surgery With Loop-Assisted Peritoneal Inversion Closure (SILS-LAPIC) for Strangulated Obturator Hernia With Small Bowel Necrosis

**DOI:** 10.7759/cureus.113432

**Published:** 2026-07-27

**Authors:** Sodai Arai, Taiki Masuda, Yusuke Yatabe, Misuzu Yamato, Mikito Inokuchi

**Affiliations:** 1 Department of Surgery, Japanese Red Cross Musashino Hospital, Tokyo, JPN

**Keywords:** acutely irreducible hernias, lapic, loop-assisted peritoneal inversion closure, obturator hernias, sils, sils-lapic, single-incision laparoscopic surgery, strangulated hernia

## Abstract

Obturator hernia is a rare pelvic hernia that predominantly affects elderly, thin women and often presents as small bowel obstruction requiring emergency surgery. Although mesh repair reduces recurrence, placing mesh in a potentially contaminated field may be inadvisable when the acutely irreducible bowel is necrotic. We report an 87-year-old woman with an acutely irreducible right obturator hernia causing small bowel obstruction; intraoperative findings confirmed strangulation with focal bowel necrosis. Emergency repair was performed through a single-incision laparoscopic surgery (SILS) approach. The bowel was reduced using a water-jet technique, and mesh was not used because of the focal necrosis. The right obturator defect was closed by peritoneal inversion and ligation with a pre-tied ligature loop (Endoloop; Ethicon, Somerville, NJ, USA), a mesh-free technique termed loop-assisted peritoneal inversion closure (LAPIC); an occult left obturator hernia was closed prophylactically using the same technique. We designate this combined strategy SILS-LAPIC. The affected bowel was exteriorized through the same umbilical incision, allowing direct visual assessment and limited wedge resection of the necrotic segment. Operative time was 64 minutes, and blood loss was 1 mL. The patient resumed oral intake on postoperative day 2 and was discharged on day 5 without complications, including obturator nerve palsy, with no recurrence at three months. SILS-LAPIC may be a feasible emergency option for selected patients with strangulated obturator hernia and limited bowel necrosis.

## Introduction

Obturator hernia is rare, accounting for less than 1% of abdominal wall hernias, but it is clinically important because delayed diagnosis can lead to bowel strangulation, necrosis, and high morbidity in frail elderly patients [1,2]. It typically occurs in elderly, thin, multiparous women and often presents with non-specific symptoms of small bowel obstruction rather than a palpable groin mass [1,3]. Computed tomography (CT) is therefore central to early diagnosis and operative planning.

Surgical repair is the definitive treatment for acutely irreducible obturator hernia. Open laparotomy has traditionally been used in emergency cases, especially when bowel ischemia is suspected, but laparoscopic approaches are increasingly reported because they allow diagnosis, reduction, bowel viability assessment, and inspection of the contralateral myopectineal orifice [3,4]. Among laparoscopic options, peritoneal closure without mesh can be useful in strangulated cases because it provides direct access to the abdominal cavity while avoiding prosthetic material [2,4]. Mesh repair is commonly used when contamination is absent, but bowel necrosis or bowel resection may influence the decision to avoid synthetic material.

Single-incision laparoscopic surgery (SILS) may further reduce surgical trauma and may contribute to early postoperative recovery in elderly patients. One method of peritoneal closure involves grasping the peritoneum, inverting it, and ligating its base with a pre-tied ligature loop. This technique has been established for defect closure in laparoscopic inguinal hernia repair [5] and is referred to here as loop-assisted peritoneal inversion closure (LAPIC). SILS repair of obturator hernia has been reported using a totally extraperitoneal approach with mesh in elective settings [6,7]; however, to our knowledge, LAPIC has not been applied to obturator hernia, nor has a single-incision, mesh-free, transabdominal repair been described for a strangulated obturator hernia with necrotic bowel. We designate this combined strategy - LAPIC performed through a SILS approach - as SILS-LAPIC, and present a case of strangulated obturator hernia with focal small bowel necrosis successfully treated by emergency SILS-LAPIC with limited wedge resection through the same umbilical incision. Informed consent was obtained from the patient for publication of this case report.

## Case presentation

Patient presentation and initial evaluation

An 87-year-old Japanese woman presented to the emergency department with abdominal pain that had started the previous day and was followed by vomiting. The patient was thin, with a body mass index (BMI) of 17.6 kg/m^2^ (height, 147 cm; weight, 38 kg) and a history of two childbirths. Past medical history included cerebral infarction and a left proximal femoral fracture. The history of abdominal surgery consisted only of appendicitis. The patient’s activities of daily living were limited; although she was able to walk with a walker, assistance was required for bathing, and she had been certified at the support-required level under Japan's Long-Term Care Insurance system.

The patient’s vital signs were as follows: body temperature 37.6°C, blood pressure 105/78 mmHg, and pulse rate 101 beats per minute. Physical examination revealed right lower abdominal tenderness, and a dilated bowel loop was detected. Laboratory tests showed a white blood cell (WBC) count of 11,900/µL, lactate dehydrogenase (LDH) 265 U/L, C-reactive protein (CRP) 0.60 mg/dL, and a mild increase in inflammatory response (Table 1).

**Table 1:** Laboratory data WBC: white blood cell; HGB: hemoglobin; CRP: C-reactive protein; LDH: lactate dehydrogenase; AST: aspartate aminotransferase; ALT: alanine aminotransferase; T-bil: total bilirubin; BUN: blood urea nitrogen; Na: sodium; K: potassium; Cl: chloride

Test (unit)	Admission value	Reference range
WBC (x10^3^/μL)	11.9	3.3-8.6
HGB (g/dL)	13.3	11.6-14.8
Platelets (x10^3^/μL)	156	158-348
CRP (mg/dL)	0.60	<0.14
Total protein (g/dL)	6.1	6.6-8.1
Serum albumin (g/dL)	3.6	4.1-5.1
LDH (U/L)	265	124-222
AST (IU/L)	31	13-30
ALT (IU/L)	20	7-23
T-bil (mg/dL)	1.1	0.4-1.5
BUN (mg/dL)	28.6	8-20
Serum creatinine (mg/dL)	0.52	0.46-0.79
Na (mEq/L)	137	138-145
K (mEq/L)	4.1	3.6-4.8
Cl (mEq/L)	102	101-108

Imaging findings

**Figure 1:**
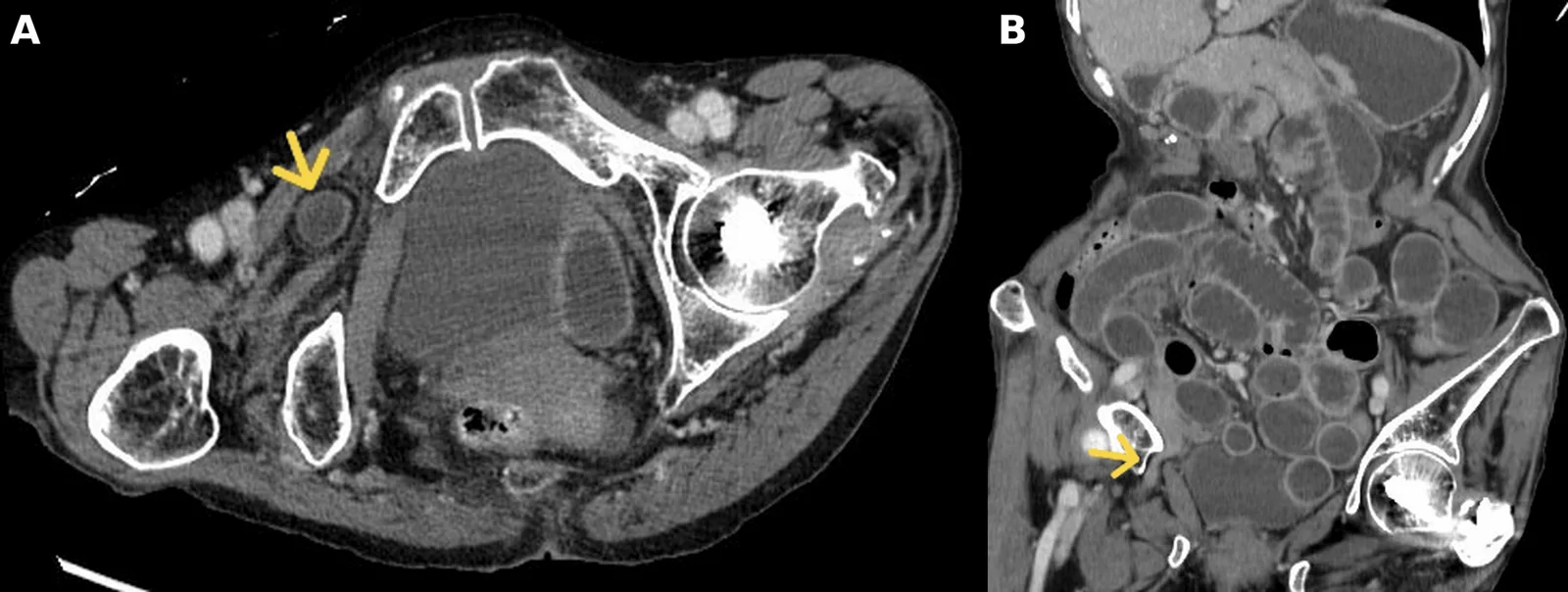
Contrast-enhanced abdominal computed tomography (CT) of the pelvis Axial (A) and coronal (B) contrast-enhanced CT images of the pelvis showing a small bowel incarceration within the right obturator foramen (yellow arrows), consistent with a right obturator hernia.

Contrast-enhanced abdominal CT demonstrated a strangulated small bowel hernia at the right obturator foramen with marked dilatation of the proximal small bowel (Figure 1). The hernia orifice measured 7 mm in maximum diameter. Contrast enhancement of the affected bowel wall was preserved, and no obvious signs of necrosis were observed. Based on these findings, the patient was diagnosed preoperatively with a strangulated right obturator hernia causing small bowel obstruction, and emergency surgery was planned. Given the patient’s advanced age, frailty, and low activities of daily living, a minimally invasive single-incision laparoscopic approach was selected to promote early postoperative recovery while allowing intra-abdominal evaluation.

Surgical technique

**Figure 2:**
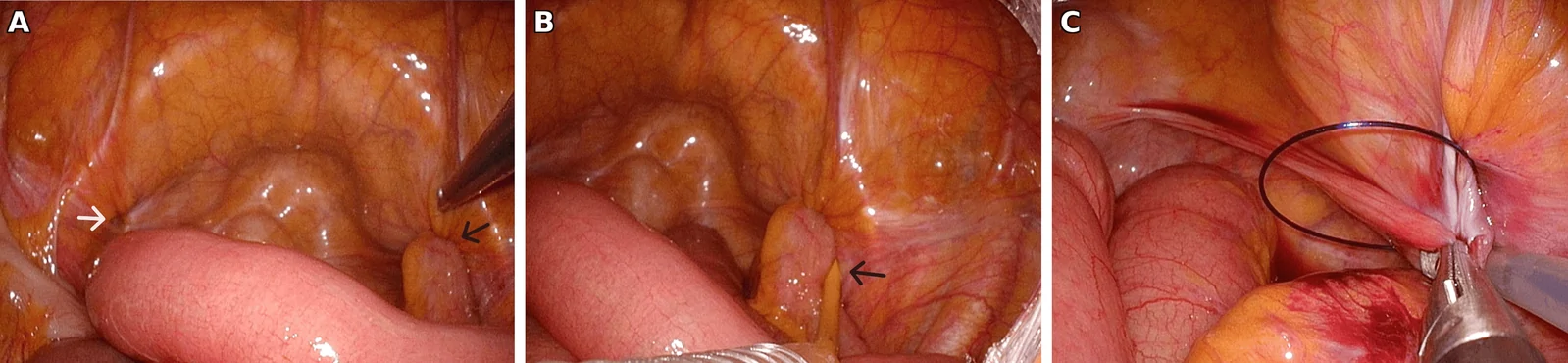
Intraoperative laparoscopic findings, reduction technique, and peritoneal closure (A) Acutely irreducible small bowel within the right obturator foramen (black arrow) and occult left obturator hernia (white arrow). (B) Reduction using the water-jet technique (black arrow: Nelaton catheter). (C) Peritoneal closure of the obturator defect.

A 2 cm umbilical incision was made extending to the umbilical ring, and the peritoneal cavity was entered by an open technique. A small wound protector with an inner diameter of 3.5 cm combined with a silicone rubber cap was placed, and three 5 mm ports were inserted through the single-incision access site. Laparoscopy revealed acutely irreducible small bowel within the right obturator foramen and an occult left obturator hernia (Figure 2A).

**Figure 3:**
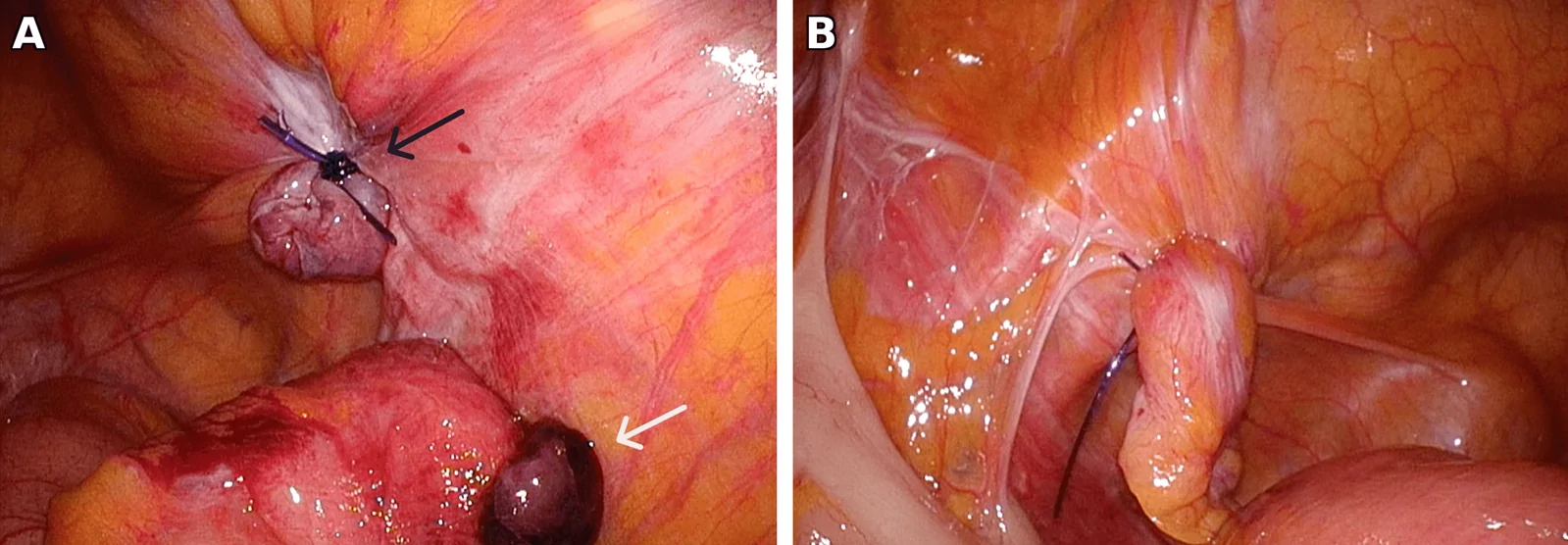
Findings after repair and assessment of reduced small bowel (A) Right-sided peritoneal closure (black arrow) and focal small bowel necrosis (white arrow). (B) Left-sided prophylactic peritoneal closure.

A 6 Fr Nelaton catheter was inserted into the right obturator defect, and the acutely irreducible bowel was reduced using a water-jet technique (Figure 2B). After reduction, a focal area of discoloration was observed on the affected bowel segment, raising concern for partial necrosis. Since the possibility of bowel resection was considered, mesh repair was not performed. The right obturator defect was closed by inverting the peritoneum and ligating it with a pre-tied ligature loop (Endoloop; Ethicon, Somerville, NJ, USA) (Figure 2C). The left obturator defect was also closed prophylactically using the same peritoneal closure technique (Figure 3).

After peritoneal closure, the affected small bowel was exteriorized through the single umbilical incision. Direct visual assessment showed that the ischemic change was confined to a focal area on the antimesenteric side, involving only part of the circumference of the bowel wall, whereas the mesenteric border and the remaining circumference appeared viable, consistent with a Richter-type strangulation. Because the involved area was circumferentially limited, segmental resection was not required; instead, a 2.5 cm full-thickness wedge resection including the discolored area was performed. The operation was completed after confirming hemostasis and bowel viability. Operative time was 64 minutes, and blood loss was 1 mL.

Postoperative course

The postoperative course was uneventful. The patient passed flatus on postoperative day (POD) 1 and resumed oral intake on POD 2. The patient recovered without obturator nerve palsy, surgical-site infection, ileus, or recurrent obstruction and was discharged on POD 5. At three months after surgery, no recurrence has been observed.

## Discussion

This case highlights three clinically relevant points. First, SILS-LAPIC provided the advantages of laparoscopy in an emergency obturator hernia: direct confirmation of the acutely irreducible small bowel, minimally invasive reduction, assessment of bowel viability, and identification of a contralateral obturator hernia. These features are consistent with prior reports supporting laparoscopic repair for selected acutely irreducible or strangulated obturator hernias [3,4,8]. Recent comparative data also suggest that laparoscopic repair may shorten hospital stay and reduce postoperative morbidity compared with open repair in selected patients, although the evidence remains limited due to small sample sizes and selection bias [9,10]. Obturator hernia has several disease-specific features that make this single-incision, mesh-free strategy particularly suitable: it predominantly affects frail, elderly, low-body-mass women in whom reduced surgical trauma and early recovery are especially valuable; it frequently presents with strangulation, so reliable assessment of bowel viability is essential; the obturator defect is small, so a mesh is often unnecessary and simple tissue closure suffices; and occult contralateral defects are common and can be addressed in the same setting. SILS repair of obturator hernia has previously been reported, but as a totally extraperitoneal approach with mesh placement in elective settings [6,7]. Because an extraperitoneal approach does not allow observation of the abdominal cavity, it is not suited to strangulated cases in which bowel viability must be assessed. To our knowledge, a transabdominal, mesh-free repair completed through a single umbilical incision has not been described for emergency obturator hernia with necrotic bowel.

Second, the single-incision access site was useful not only for laparoscopy but also for direct visual assessment of the small bowel. Because strangulation is common in obturator hernia, reliable evaluation of bowel viability is critical, and the decision between observation, limited resection, and segmental bowel resection can be difficult when ischemic change is localized. Laparoscopic-assisted strategies with mini-laparotomy have been described as a practical way to combine minimally invasive diagnosis and reduction with safe extracorporeal bowel resection when necrosis is present [11]. A particular advantage of the single-incision approach is that the umbilical access site itself served as the extraction window: the strangulated small bowel was exteriorized through the same incision for direct visual assessment and limited wedge resection, without an additional incision. Because obturator hernia is frequently of the Richter type, the ischemic change is often confined to part of the circumference on the antimesenteric side. In such cases, a full-thickness wedge resection of the involved area removes the entire thickness of the compromised wall while preserving bowel length and avoiding an anastomosis, which is advantageous in frail elderly patients. This preserved the minimally invasive nature of the procedure while ensuring safe treatment of the necrotic tissue.

Third, non-mesh repair was selected because focal bowel necrosis required resection. Mesh repair may reduce recurrence and is widely used when the operative field is clean; previous literature suggests lower recurrence after mesh repair than non-mesh repair, and some reports describe successful mesh use even with bowel resection when contamination was strictly controlled [4,9,12]. Nevertheless, in a frail elderly patient with necrotic bowel, avoiding synthetic material was considered prudent. Based on the same principle of avoiding synthetic materials in areas at risk of contamination, Miki et al. used staples instead of mesh to close the defect in a case of strangulated obturator hernia requiring bowel resection [13]. Peritoneal closure or flap-based non-mesh repair has likewise been reported in strangulated obturator hernia when bowel ischemia or perforation makes synthetic reinforcement undesirable [14]. LAPIC itself is not a new surgical technique: inversion of the peritoneum and ligation of its base with a pre-tied ligature loop has been established for primary closure of the direct inguinal hernia defect during laparoscopic repair, where it is used mainly to obliterate the pseudosac and reduce seroma [5]. The novelty of the present case lies not in the technique but in its application: in obturator hernia, the small defect (7 mm in the present case) makes mesh reinforcement often unnecessary, so LAPIC can serve as the definitive, mesh-free closure rather than an adjunct, and its combination with a single-incision approach (SILS-LAPIC) allows the entire repair and direct visual bowel assessment to be completed through one umbilical incision. To our knowledge, this application to obturator hernia has not been previously described; prior non-mesh closures of the obturator defect have relied mainly on staples or sutures [13]. On the other hand, since non-mesh repair may carry a risk of recurrence, careful follow-up is necessary. In our limited experience, we have performed LAPIC in a small number of similar cases without observing recurrence to date; however, the number of patients and the length of follow-up remain limited, and further accumulation of cases is needed to determine the long-term durability of SILS-LAPIC.

This case also emphasizes the importance of examining the contralateral obturator foramen. Laparoscopy offers a broad view of the pelvis, and occult bilateral obturator hernias are not uncommon in frail elderly patients. In one laparoscopic series, occult hernias, including contralateral obturator hernias, were identified in 60% of patients and repaired during the same operation [8]. Prophylactic closure of the left-sided defect was performed to reduce the risk of future acute irreducibility while avoiding mesh placement.

This report has limitations. It describes a single patient with localized necrosis and minimal contamination; therefore, the outcome may not apply to patients with diffuse peritonitis, perforation, hemodynamic instability, or extensive bowel ischemia. In addition, although CT showed no obvious contrast defect, focal bowel ischemia was identified intraoperatively. This discrepancy may be partially explained by the approximately three-hour interval between CT imaging and surgery, during which bowel ischemia could have progressed. The optimal repair method in contaminated emergency obturator hernia remains individualized, depending on bowel viability, contamination, defect size, patient frailty, and surgeon experience.

## Conclusions

SILS-LAPIC is a feasible mesh-free option for strangulated obturator hernia complicated by bowel necrosis. Because the obturator defect is small, LAPIC may constitute a definitive repair rather than an adjunctive procedure, even in a potentially contaminated field. A transabdominal single-incision approach additionally allows bowel viability to be assessed under direct vision, which is not possible with an extraperitoneal repair.

We suggest that SILS-LAPIC be considered in frail elderly patients when contamination makes mesh placement undesirable. Because non-mesh repair may carry a risk of recurrence, long-term follow-up and further accumulation of cases are required.
